# Cervical cancer screening decentralized policy adaptation: an African rural-context-specific systematic literature review

**DOI:** 10.1080/16549716.2019.1587894

**Published:** 2019-04-02

**Authors:** R. Rahman, M. D. Clark, Z. Collins, F. Traore, E. M. Dioukhane, H. Thiam, Y. Ndiaye, E. L. De Jesus, N. Danfakha, K. E. Peters, T. Komarek, A. M. Linn, P. E. Linn, K. E. Wallner, M. Charles, M. Hasnain, C. E. Peterson, J. A. Dykens

**Affiliations:** a University of Toledo College of Medicine and Life Sciences, Toledo, OH, USA; b Library of the Health Sciences, University Library, University of Illinois at Chicago, Chicago, IL, USA; c Department of Medical Education, College of Medicine, University of Illinois at Chicago, Chicago, IL, USA; d Department of Family Medicine, Institute for Health Research and Policy, University of Illinois at Chicago (UIC), Chicago, IL, USA; e Region medical de Kedougou, Bureau de la santé de la reproduction, prevention transmission mere enfant du VIH-SIDA, Kedougou, Senegal; f Senegal Ministry of Health; g Region medical de Kedougou, Bureau régional de la formation, de la supervision et de la recherche, Kedougou, Senegal; h Département de Recherche, Sénégal Ministère de la Santé et l‘Action Sociale, Dakar, Sénégal; i School of Public Health (SPH), University of Illinois at Chicago (UIC), Chicago, IL, USA; j John Snow, Inc, Arlington, VA, USA; k School of Public Health, Division of Community Health Sciences, Illinois Prevention Research Center, Institute for Health Research and Policy, University of Illinois at Chicago (UIC), Chicago, IL, USA; l Uniformed Services University, Bethesda, MD, USA; m School of Public Health, Center for Communications Programs, Johns Hopkins University, Baltimore, MD, USA; n Logistics Management Institute, Tysons, VA, USA; o Elizabeth Glaser Pediatric AIDS Foundation, Washington, DC, USA; p Department of Internal Medicine, University of Illinois at Chicago, Chicago, IL, USA; q Department of Family Medicine, University of Illinois at Chicago College of Medicine, Chicago, IL, USA; r School of Public Health, Division of Epidemiology & Biostatistics, UI Cancer Center, University of Illinois at Chicago (UIC), Chicago, IL, USA; s Department of Family Medicine, Center for Global Health, Institute for Health Research and Policy, Cancer Center, University of Illinois at Chicago (UIC), Chicago, IL, USA

**Keywords:** Implementation, partnership, global health, visual inspection of the cervix with acetic acid, gynecologic cancer

## Abstract

**Background**: Worldwide, nearly 570,000 women are diagnosed with cervical cancer each year, with 85% of new cases in low- and middle-income countries. The African continent is home to 35 of 40 countries with the highest cervical cancer mortality rates. In 2014, a partnership involving a rural region of Senegal, West Africa, was facing cervical cancer screening service sustainability barriers and began adapting regional-level policy to address implementation challenges.

**Objective**: This manuscript reports the findings of a systematic literature review describing the implementation of decentralized cervical cancer prevention services in Africa, relevant in context to the Senegal partnership. We report barriers and policy-relevant recommendations through Levesque’s Patient-Centered Access to Healthcare Framework and discuss the impact of this information on the partnership’s approach to shaping Senegal’s regional cervical cancer screening policy.

**Methods**: The systematic review search strategy comprised two complementary sub-searches. We conducted an initial search identifying 4272 articles, then applied inclusion criteria, and ultimately 19 studies were included. Data abstraction focused on implementation barriers categorized with the Levesque framework and by policy relevance.

**Results**: Our findings identified specific demand-side (clients and community) and supply-side (health service-level) barriers to implementation of cervical cancer screening services. We identify the most commonly reported demand- and supply-side barriers and summarize salient policy recommendations discussed within the reviewed literature.

**Conclusions**: Overall, there is a paucity of published literature regarding barriers to and best practices in implementation of cervical cancer screening services in rural Africa. Many articles in this literature review did describe findings with notable policy implications. The Senegal partnership has consulted this literature when faced with various similar barriers and has developed two principal initiatives to address contextual challenges. Other initiatives implementing cervical cancer visual screening services in decentralized areas may find this contextual reporting of a literature review helpful as a construct for identifying evidence for the purpose of guiding ongoing health service policy adaptation.

## Background

In 2018, globally 569,847 cervical cancer diagnoses and 311,000 deaths were projected due to this preventable disease [1]. Cervical cancer is the fourth most common cancer diagnosed among women worldwide. It has the highest cancer incidence rate among women in 28 countries and is the most common type of cancer-related mortality among women in 42 countries, the majority being in sub-Saharan Africa [1]. While cervical cancer incidence rates are declining in high-resource areas, incidence, prevalence, and mortality rates continue to rise in low- and middle-income countries (LMICs) [1]. Furthermore, cervical cancer mortality is expected to increase by 42% to 442,926 deaths in the year 2030 [2]. The greatest rise will be in LMICs where currently 85% of incident cervical cancers and 87% of cervical cancer deaths occur [3,4].

### Cervical cancer screening

Various evidence-based cervical cancer screening techniques have been developed and tested, and are appropriate for diverse contexts including (a) cytologic screening through Papanicolaou (Pap) smear with follow-up colposcopy and biopsy to identify early stage dysplasia and pre-cancers, (b) human papillomavirus (HPV) testing through clinician-sampled or self-sampling techniques, and (c) visual inspection methods (employing acetic acid and/or Lugol’s solution), which are effective, low-cost approaches appropriate for low-resource settings. Visual inspection methods can complement other screening modalities and have been shown to have adequate sensitivity and specificity to identify later stage pre-cancers. Visual inspection of the cervix with acetic acid (VIA) is the most common screening approach implemented in resource-limited settings in LMICs [5]. VIA is performed by applying a vinegar solution to the cervix followed by a ‘naked-eye’ visual inspection to identify precancerous lesions which are then treated through freezing or the loop electrosurgical excision procedure (LEEP). The VIA screening test has a sensitivity of 82.4% (76.3% to 87.3%) and a specificity of 87.4% (77.1% to 93.4%) [6], and has been proven to be safe and cost effective [7–9]. However, despite the many screening modalities appropriate for low-resource contexts, cervical cancer screening coverage, globally, remains insufficient. Wide disparities exist between countries, as illustrated by Austria (effective screening coverage above 80%) and Ethiopia (less than 1%). This type of disparity contributes to an average coverage rate of 36.9% globally and only 18.5% in less-developed countries [10]. The contextual consideration of policy development and the adaptation of these policies to changes over time is critically important for achieving community access to high-quality health services globally [11–15].

### Decentralized cervical cancer screening

A further challenge to cervical cancer prevention and control is the ongoing disparity of access to cervical cancer services between urban and rural settings within countries [16]. Centralized policy often dictates how decentralized settings are governed and supported financially, through capacity management (personnel placement and trainings). Policies would ideally reflect the differences in context between areas with various levels of rurality and development. Evidence is necessary to inform the creation and refinement of these policies. However, the 10/90 gap, a global health disparity defined as less than 10% of global funding for research being spent on health issues that afflict more than 90% of the population, continues to plague the world’s most marginalized communities [17]. This has considerable implications in sub-Saharan Africa and, in particular, for women’s health issues [18,19]. Hence, there remains a paucity of literature describing the implementation, strengthening, and sustainment of cervical cancer prevention and control programs in Africa. Given that decentralized health systems, especially in rural, resource-limited settings, have made some progress toward sustaining accessible screening services and reducing disparities in cervical cancer rates, it is imperative to gain a better understanding of the context in which cervical cancer prevention and control programs are implemented effectively and sustained.

### Cervical cancer prevention and control policy relevance

Local health service practice guidelines and regional health systems policy ideally address the most common implementation barriers within a given context in order to optimize impact as well as ensure program sustainability. Factors such as acceptability, adoption, appropriateness, and feasibility are critical health service implementation components of ensuring sustainable, high-quality, accessible, person-centered health care services within a community health system [13]. Health systems that employ dynamic thinking, systems-as-cause consideration, context analysis, operational thinking, and loop thinking (i.e. viewing causality as an ongoing process) will make more impactful strides in programmatic and policy planning [20]. Cervical cancer is also a critical indicator of larger health system challenges including poor access to quality primary health care services and the lack of culturally competent communication – both factors that disproportionately affect low-income women [21]. As such, cervical cancer prevention and control, both at the programmatic level of a single health care service delivery structure, and at the elevated global population-based level, requires comprehensive systems thinking [20,22]. The assurance of quality and the long-term sustainment of newly implemented cervical cancer screening programs necessitate considerable attention to systems-level factors beyond workforce capacity building.

### Cervical cancer partnership in Senegal

The above considerations have figured prominently in the development and advancement of collective action within a partnership formed in Senegal. Global health partnerships take many forms and can include various partners including health ministries, academic institutions, and non-governmental organizations (NGOs), and have become the dominant organizational model, globally, to address many health systems challenges globally [23]. In 2010, the Kedougou Medical Region in Southeastern Senegal, Peace Corps Senegal, the Institute of Health and Development at Cheikh Anta Diop University, Dakar, Senegal, and the University of Illinois at Chicago (UIC) formed a partnership with the purpose of improving community access to quality primary health care services. The cervical cancer incidence rate in Senegal (41.1%) places it 15^th^ in the world in the age-standardized incidence rate of cervical cancer [24]. Between 2010 and 2013, the partnership worked to build capacity across Kedougou to ensure cervical cancer VIA screening access to an estimated 9041 women in the targeted age group (30 to 50 years old, at the time) [25–27]. Despite robust community-level education, screening rates were suboptimal, with less than 5% of the population being screened in some locations [28]. A 2014 study concluded that only 38% of women in the study area were aware of cervical cancer, highlighting lack of awareness as a major barrier to women accessing screening services. Despite efforts to target the full age range of at-risk women, the mean age was relatively young (35.7) for the total sample [28]. Furthermore, the highest risk older cohort of women were the ones least likely to seek screening services [28]. Based on our findings it was evident that a standard, non-context-specific, unadapted approach to raising cervical cancer prevention awareness in this region was inadequate.

In 2014, we undertook a literature review to identify program evaluation or policy-relevant publications with a focus on settings similar to the context of this rural Senegal region. The intent was both to improve uptake of cervical cancer screening in this region and to inform the future horizontal scale of the VIA screening program to neighboring rural regions by shaping informed, context-specific regional health policy. Concurrently, the Senegal National Ministry of Health and Social Action initiated an effort to develop a National Cancer Control and Prevention Plan that includes cervical cancer screening implementation in the rural zones as an explicit priority. This systematic literature review reports on research describing the implementation of cervical cancer prevention services at the decentralized level in Africa. We analyzed the literature to identify cervical cancer health services implementation challenges and proposed solutions in very similar contexts to that of the Kedougou, Senegal, partnership. We organized these identified barriers and policy-relevant recommendations according to the Patient-Centered Access to Healthcare Framework proposed by Levesque [29]. The aim of this review was to inform ongoing cervical cancer screening health services implementation in Senegal to enhance the quality of services offered, increase utilization among communities, and ensure the sustainability of health services. In this report, we describe the findings of the systematic review and illustrate how the Senegal partnership utilized this approach to adapt regional policy in developing next-phase initiatives as a response to ongoing implementation barriers [30]. Sub-searches 1 and 2 were merged within each database (see Appendix A for the PubMed search strategy).

## Methods

We conducted a systematic review of the electronic literature published on cervical cancer screening using visual inspection methodology in African nations according to the Preferred Reporting Items for Systematic Reviews and Meta-Analyses (PRISMA) guidelines [31]. PubMed (1950 to 11/30/14), EMBASE (1974 to 12/1/14), CINAHL (1937 to 12/1/14), and ISI Web of Science (1950 to 12/1/14) were searched.

The search strategy comprised two complementary sub-searches, including controlled vocabulary and keywords. Sub-search 1 intersected three concept sets: (a) screening terms (e.g. mass screenings, early detection of cancer, preventive health services, primary prevention, testing, HPV testing, smears, gynecological examination, obstetric and gynecological diagnostic techniques, health promotions, early interventions); (b) cervix uteri terms (e.g. cervix, cervix uteri); and (c) African nations and ‘low-resource settings’ terms (e.g. Africa, Algeria, Malawi, low-resource nations, low-resource regions, underdeveloped countries, poorest countries). Sub-search 2 intersected four concept sets: (d) cervix uteri terms (as above); (e) cancer/pre-cancer terms (e.g. cancers, malignancies, dysplasias, lesions, abnormalities, adenocarcinomas, precursors, warts, tumors); (f) visual inspection terms (e.g. acetic acid, iodine, Lugol’s, visual inspection, visual evaluation); and (g) cervical vertebra terms together with terms to exclude animals (e.g. NOT neck, cervical atlas, root caries, thyroid, rats, cats, dogs). An English-only filter was applied after the results of sub-searches 1 and 2 were merged.

Selected studies met all inclusion criteria: (a) the article reported a clinical study or outlined clinical guidelines or policy; (b) visual inspection for cervical cancer screening was focal; (c) the study was set at a decentralized level (regional, district, village) African LMIC. Excluded studies met at least one of the following exclusion criteria: (a) the study included mixed population data (e.g. populations outside of an African LMIC); (b) the study was strictly epidemiologic and without clinical relevance; and (c) the study described screening tool effectiveness without reporting other clinically relevant findings.

**Figure 1.**
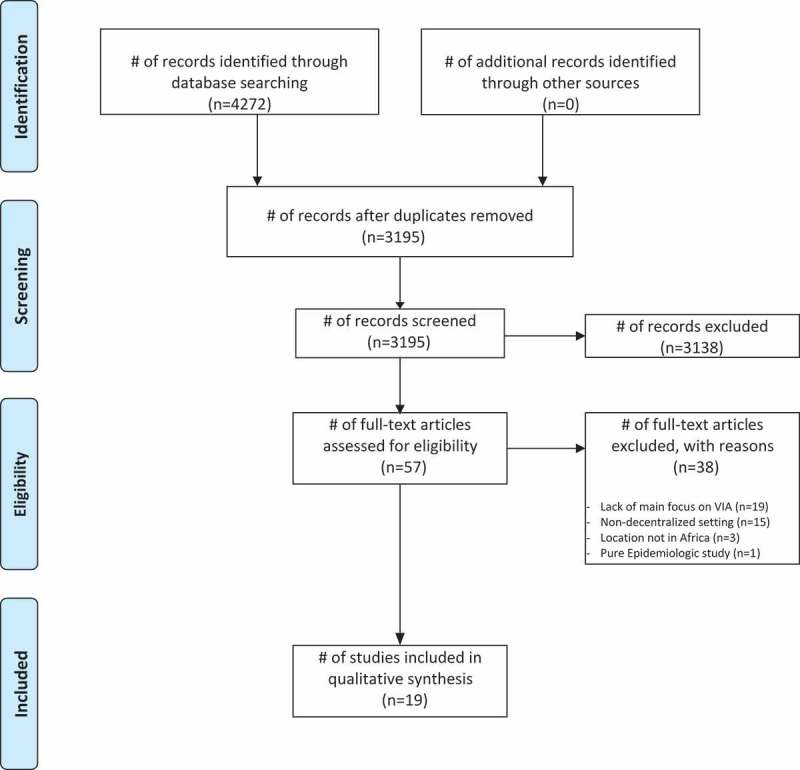
PRISMA flow diagram.

A total of 4272 citations were retrieved from the database searches and 3195 citations remained after deduplication. The titles and abstracts of these citations were screened for relevance by teams of two collaborators, and 57 full-text articles were subsequently evaluated according to the inclusion criteria. Selection of studies based on application of the inclusion criteria was accomplished by teams of two collaborators by consensus, with the principal investigator making the final determination in the event of disagreement. Nineteen studies met the inclusion criteria and were included in the review. The PRISMA flow diagram in Figure 1 shows the steps taken for article selection.

### Data abstraction and synthesis

In order to elucidate common themes from each text, a data abstraction tool in the form of a Data Abstraction Sheet was created by the authors. The data abstraction tool was piloted by two reviewers and edited with feedback. A single, final abstraction tool was used for all included articles. This abstraction tool allowed two separate reviewers to independently read each article and abstract pertinent data without personal interpretation. The group members were allowed to give feedback on the categories and abstraction tool to help refine the Data Abstraction Sheet and create a better understanding of the categories. Abstracted categories include: (a) partnership type, (b) level of studied intervention (demand-side or supply-side), (c) barriers to access, and (d) descriptive (purpose, methodology, major findings). The ‘barriers’ category in the abstraction tool followed the Levesque Patient-Centered Access to Health Care Framework, specifying specific barriers on both the demand side (clients and community) and the supply side (health services) [29]. Demand-side barriers were subcategorized as ‘Perceive’, ‘Pay’, ‘Reach’, ‘Seek’, and ‘Engage’, while supply-side barriers were subcategorized into ‘Availability and Accommodation’, ‘Approachability’, ‘Affordability’, ‘Acceptability’, and ‘Appropriateness’. The abstraction categories are shown in more detail in Tables 1 and 2.

**Table 1.** Description of studies (n = 19).

	n	%
**Type of study**		
Descriptive Research	10	**53**
Correlation Study	5	**26**
Semi-Experimental Design	4	**21**
Experimental Design	0	**0**
Policy	0	**0**
**Years published**		
2010 - 2015 ** **	17	**89**
2005 - 2009	2	**11**
2000 - 2004	0	**0**
<2000	0	**0**
**Location of Study - 8 unique African countries**		
Nigeria	5	**26**
Malawi	2	**11**
Mozambique	2	**11**
Tanzania	2	**11**
Zambia	2	**11**
Ghana	1	**5**
Uganda	1	**5**
Zimbabwe	1	**5**
Study Includes Multiple Countries of Interest (at least one African country)	3	**16**
Ghana and Thailand	1	5*
Uganda and El Salvador	1	5*
Uganda, Peru, and Vietnam	1	5*
**Country Income Groups According to World Bank**		
**Classifications**	10	**53**
Low income	9	**47**
Lower middle income	0	**0**
Upper middle income	0	**0**
High income		
**Clinical Approach**		
Visual Inspection with Cryotherapy as a Single Approach	12	**63**
Visual Inspection Only - with Cryotherapy Upon Later Visit	1	**5**
Visual Inspection Only - No Explicit Link to Cryotherapy	6	**32**

* Percentage is reflective of the three articles within the ‘Multiple countries of interest’ category.

**Table 2.** Partnerships table.

		Mangoma 2006	Sanghvi 2008	Peters 2010	Fort 2011	Levine 2011	Mwanahamuntu 2011	Quentin 2011	Audet 2012	Moon 2012	White 2012	Adetokunbi 2013	Chigbu 2013	Paul 2013	Perng 2013	Abiodun 2014	Chigbu 2014	Chigbu 2014	Maseko 2014	Osingada 2014	Total
Partnership Type	Academic institution - International			x	x	x	x	x		x	x			x	x					x	**10**
	Academic institution - National	x	x			x	x	x		x		x				x		x	x	x	**11**
	NGO - International		x		x	x			x			x									**5**
	NGO - Local									x		x			x						**3**
	Government / Health system - International						x		x					x							**3**
	Government / Health system - Local	x	x	x	x		x	x	x	x	x	x	x	x	x						**13**
	Government / Health system - National	x	x			x	x	x	x	x							x			x	**9**
	**Total:**	**3**	**4**	**2**	**3**	**4**	**5**	**4**	**4**	**5**	**2**	**4**	**1**	**3**	**3**	**1**	**1**	**1**	**1**	**3**	

Based on the number of mentions, the top four policy-relevant demand- and supply-side barriers from the literature review are presented. We also identified and summarize policy recommendations contained within the selected literature relevant to the rural African context. All articles referring to a specified barrier or policy recommendation are cited for the category, but only select references are included to illustrate specific barriers or policy recommendations. Other papers within the sample may have included comments of a similar spirit.

## Results

The search identified 19 research papers focused on visual inspection screening for cervical cancer at a decentralized level in an LMIC. The research designs of the included articles are: descriptive research (e.g. observational, case study, or survey; n = 10, 53%), correlation study (eg. case control, cohort, cross-sectional; n = 5, 26%), and semi-experimental design (eg. field experiment, twin study; n = 4, 21%). There are no studies that employed experimental design, and there are no policy papers included. Two (11%) of the papers were published between 2005 and 2009 while 17 (89%) were published between 2010 and 2015. The studies took place in eight unique African countries: Ghana, Malawi, Mozambique, Nigeria, Tanzania, Uganda, Zambia, and Zimbabwe. Nigeria was the most studied location, being mentioned in five (26%) different papers, while only three (16%) of the papers include multiple study locations. Nine (47%) papers focus on countries classified as lower middle income by the World Bank, while 10 (53%) countries are classified as low income [32]. Three main clinical approaches of ‘see and treat’ were used in the articles: visual inspection with cryotherapy as a single approach (n = 12, 63%), visual inspection only with referral for cryotherapy (n = 1, 5%), and visual inspection only with no referral (n = 6, 32%). See Table 1 for a description of the studies.

All included articles report projects that were managed through global health partnerships involving various partner types. Of these, 11 (58%) of the partnerships included within-country academic institutions, 10 (53%) included international academic institutions, and 13 (68%) had involvment of local (decentralized) government or formal health systems (Table 2).

Our findings identified specific barriers on both the demand side (clients and community) and the supply side (health services). Using the Levesque Patient-Centered Access to Health Care Framework, we identified 17 (89%) articles that report demand-side barriers. Most of these articles report barriers across multiple categories specified in the Levesque framework: ‘Perceive’ is reported in 13 (76%) articles, ‘Pay’ in 12 (71%), ‘Reach’ in 11 (65%), ‘Seek’ in ten (59%), and ‘Engage’ in nine (53%) (Table 3). Seventeen (89%) report supply-side barriers, with 14 (82%) articles reporting barriers relevant to ‘Availability and Accommodation’. ‘Approachability’ is discussed in eight (47%) articles reporting supply-side barriers, ‘Affordability’ in six (35%), ‘Acceptability’ in three (18%), and ‘Appropriateness’ in three (18%) (Table 4).

**Table 3.** Articles reporting demand-side barriers to access (n = 17).

	Articles		Mentions
	n	%	n = 62
**Perceive**	13	76	
*Health Llteracy –* Patient awareness, knowledge, or education deficiency			12
*Beliefs related to health and sickness –* Pain or discomfort during the procedure			4
*Trust and expectations –* Distrust of screening methodology or efficacy or quality			4
*Beliefs related to health and sickness –* Side effects on sexual performance			2
*Beliefs related to health and sickness –* Stereotyped risk			1
**Seek**	10	59	
*Personal and social values –* Permission required from family/lacking social support			9
*Gender –* Embarrassment or anxiety in the clinical setting			4
*Culture –* Belief that cervical cancer is a curse by foreigners/witchcraft			2
**Reach**	11	65	
*Geographic reach –* Access challenges (e.g. multiple visits, distance)			9
*Occupational flexibility –* Do not have time			5
*Living environment –* Drought			1
**Pay**	12	71	
*Income vs poverty –* Inability to pay (significant cost to the patient)			12
**Engage**	9	47	
*Self-management –* Preventive care low priority/not primary reason to visit clinic			6
*Caregiver support –* Communication challenges (encouragement, teaching mode)			4

*Categories in bold font represent the Levesque framework, categories in italic font represent Levesque framework subcategories, and categories without boldface or italics represent the specific barrier mentioned in the papers. **(n) = the number of papers reporting in this category. (%) = the percentage of 17 papers reporting demand-side barriers. (mentions) = the number of mentions in subcategories (note that one paper may include multiple subcategory mentions).

**Table 4.** Articles reporting supply-side barriers to access (n = 17).

	Articles		Mentions
	n	%	n = 62
**Approachability**	8	47	
*Outreach –* Lack of outreach			8
**Acceptability**	3	18	
*Gender of provider –* Female provider preferred but unavailable			3
**Availability and accommodation**	14	82	
*Providers –* Health workforce shortages or low daily presence of service providers			11
*Appointments, modes of service provision –* Lack of follow-up			8
*Facility characteristics –* Lack of supplies or electricity needed for screening			7
*Appointments, modes of service provision –* Long wait			4
*Providers –* Burnout and turnover			4
*Geographic location and context –* Distance or spread in rural populations			3
*Providers –* Lack of administrative oversight of screening program			3
*Facility characteristics –* Functionality of equipment			2
**Affordability**	6	35	
*Direct costs of services and related expenses* – Cost of screening			5
*Indirect costs* – Clinical space repairs and renovations			1
**Appropriateness**	3	18	
*Technical and interpersonal quality –* Lack of training programs or advancement			3

*Categories in bold font represent the Levesque framework, categories in italic font represent Levesque framework subcategories, and categories without boldface or italics represent the specific barrier mentioned in the papers. **(n) = the number of papers reporting in this category. (%) = the percentage of 17 papers reporting demand-side barriers. (mentions) = the number of mentions in subcategories (note that one paper may include multiple subcategory mentions).

### Demand-side access barriers and policy recommendations

- **Patient awareness, knowledge or education deficiency –** 12 articles [33–44].

**Barriers**: Authors note that a lack of understanding of personal risk or insufficient cervical cancer education [36] may lead to a decreased sense of urgency to seek cervical cancer screening. A commonly cited example is a woman’s misunderstanding that she is only at risk if she displays signs or symptoms or feels sick [35], but would otherwise not need to be screened. A lack of awareness about the presence of cervical cancer preventative services [35] also decreases screening uptake. Misperceptions about known services can also lead to unwarranted fears such as assumptions that the procedures used to screen or treat cervical cancer are uncomfortable or that instrument cleanliness is substandard [44]. Additionally, misunderstandings about screening and treatment may lead to stigma [35], myths [44], and misinformation [41], further impacting screening uptake.

**Recommendations**: Various methods suggested for increasing patient awareness, knowledge, and education include but are not limited to: (a) counseling sessions that incorporate educational videos [36], (b) health educators emphasizing the benefits of screening (less pain, potential protection against future cancer, and lower rates of mortality) rather than focusing on the sexual cause of the disease [34], (c) creating specific curricula targeted at men so they can help motivate and support women to increase screening utilization while improving male sentiment toward the screening [42], (d) recruiting peer educators who are at times more personable and accessible than physicians to answer follow-up questions [41], (e) using media such as the radio to build health literacy to help increase awareness and encourage women to seek out screenings or attend workshops in the area [33], and (f) implementing media-led education to increase recognition of services [36].

- **Inability to pay –** 12 articles [33–38,40–43,45,46]

**Barriers**: The economic burden on women and their families (real or perceived) greatly limits screening uptake. Examples of this burden include but are not limited to: the perceived cost of screening or treatment [33], travel expenses [37], lost wages because of missing work [42], and fear of hidden costs [43].

**Recommendations**: VIA is recommended in certain contexts over other cervical cancer screening tests, such as the Pap smear, to keep costs for the patient low; however, not all patients are able to pay for screening and so it is recommended that the health care facilities provide discounted or free screening [35]. One study noted that only 18% of women screened for free would participate if there was a fee for screening [46]. Furthermore, if more patients are screened and treated then economies of scale can potentially lower costs for future patients [45]. Cost-effectiveness [39] could be increased by creating models in clinics that offer screening services and treatment services for dysplasia in a single visit (screen and treat).

- **Geographic reach access challenges –** nine articles [35,38,40,42–47]

**Barriers**: Access to services is a common problem in rural areas with no nearby treatment centers [35,38,40,42–47], or in rural areas where the infrastructure is too deficient to support the keeping and proper care of the equipment required for comprehensive referral services [47]. If multiple visits are required for screening and treatment, responsibilities at home may create barriers to screening access or follow-up [35,38,40,42–47].

**Recommendations**: Although the papers in the literature review do not explicitly study the utilization of mobile clinics for VIA in rural areas in Africa [37], it is recommended to combat problems of access and this method should be further explored through research [38].

- **Permission required from family/lacking social support –** nine articles [33–36,38,41–44]

**Barriers**: Lack of social support from a husband [35], friends [41], or community leaders [42] may dissuade women from seeking screening services or from following up with medical professionals. In addition, cultural or social barriers [41] may cause fear of examination when there is a lack of general acceptance by the community of the benefits of cervical cancer screening.

**Recommendations**: Building relationships with organizations and local institutions can help gain rapport with influential family members [44], peers, and respected leaders [42] in the community, which may garner support for life-saving interventions [33]. Gaining trust from the local citizens may help solve logistical issues of social support [34]. Strict standards of confidential communication enable trust networks to form between medical professionals and patients and is a keystone to success by helping to eliminate worries patients may have about others’ opinions in their community. The reassurance of confidentiality will increase women’s willingness to seek medical help as a preventative measure rather than a last chance at survival [41]. To create a sustainable health service, women’s voices in the service-delivery planning must be prioritized, to the point where treatment and screening services are considered to be an investment in women.

### Supply-side barriers and policy recommendations

- **Health workforce shortages or low daily presence of service providers –** 11 articles [35,37–41,43,44,47–49]

**Principal barrier**: The number of trained health care providers (physicians, nurses, midwives, and community health workers) is critically insufficient and does not meet patient demand in many rural locations.

**Recommendations**: To combat trained service provider shortages, a five-day VIA course for health care providers was effective at teaching skills in Uganda [49]. Furthermore, the study suggested that high-quality, cost-effective standardization in VIA training curricula can help lower mortality, morbidity, and misidentification in cervical cancer while increasing the number of health care providers to help with shortages [49]. Programs should increase correspondence with other clinics in order to produce further training opportunities for local providers [38], while also creating a network that can share administrative or clinical ideas and resources to create more effective workspaces [48].

- **Lack of outreach –** eight articles [33,35,38,39,42–44,50]

**Barriers**: Without an active effort to advertise and inform the community regarding screening service availability and the necessity for prevention, women may underutilize [39] the clinical service, especially if they interpret a lack of symptoms as a sign of good health [35]. Community health systems must commit to leveraging multiple forms of media, such as focus groups and radio, to spread the message about screening services. However, given the lack of infrastructure in low-resource settings, media intervention may not be enough to reach or alert all who need care [33].

**Recommendations**: Mobile units from government hospitals can be sent out to provide screening training for health care providers at smaller hospitals [38]. Outreach can be implemented by health promoters and by women in the community who have received screening [44]. Taking a community-engaged approach, health care providers can meet with leaders of traditional institutions to advocate for screening while asking community leaders to spread awareness about appointments for screening and treatment [42]. Health care providers should also recommend screening whenever women come into the clinic regardless of their chief complaint, because women who do not receive this recommendation are 84% less likely to be screened [43]. Nurse-midwives should be utilized to encourage screening because their training in reproductive services helps foster a sense of trust and reduces anxiety in female patients [50]. Clinics could also have health care providers give reproductive health talks in villages or rural communities to encourage outreach [44].

- **Lack of follow up –** eight articles [34,35,37,42,44,46,47]

**Barriers**: Ensuring follow-up for routine screening or treatment of abnormal lesions is challenging if appointments are spaced far apart or if patients are routinely kept waiting. Without a reliable record-keeping system, health care providers may lose track of referrals or follow-up appointments [35,48]. Moreover, in low-resource settings, clinicians are often pulled away for trainings or other administrative responsibilities, further challenging timeliness of patient follow-up [48]. Follow-up exams may also be delayed if adequate materials and supplies for screenings or treatment are not readily available [35].

**Recommendations**: To increase follow-up, clinics should incorporate electronic patient tracking systems, to help eliminate poor record-keeping and repetitive data entry on paper copies that may be lost [48].

- **Lack of supplies or infrastructure needed for screening –** seven articles [35,37,38,42,44,47,48]

**Barriers**: The lack of supplies was a recurring identified barrier for initial and follow-up screenings. Poor administrative oversight for stocking supplies plays a critical role in service quality [48]. Other clinical supply shortages that were identified include lack of anesthesia to perform inspection or treatment [47] and lack of gasoline to travel to rural locations [35]. Other common health structural challenges impacting cervical cancer services in resource-poor areas include the lack of reliable electricity and suboptimal measures for the prevention of theft or damage to supplies [48].

**Recommendations**: Addressing supply-side barriers is key to growing a sustainable cervical cancer prevention service. This requires robust administrative oversight and communication with the central health system for mobilization of support and resources. Health care providers will be able to better address patient needs if their own barriers to providing service are minimized. Ensuring that local staff is monitoring supplies and re-ordering when necessary is critical. In addition, training local technicians to maintain equipment for cryotherapy so it does not malfunction and prevent treatment for long periods of time [48] ensures prevention and rapid resolution of problems.

## Discussion

Given that the intent of this literature review was to inform the implementation and strengthening of cervical cancer screening services in Kedougou, Senegal, and, ultimately, national-level cervical cancer screening policy focused on decentralized program implementation, we were forced to reconcile the fact that the literature lacks policy-specific papers focused on the decentralized low-resource settings within low-income countries in Africa. The absence of this type of knowledge sharing is problematic given the critical need to develop sustainable, efficient solutions in these settings, where context considerations are of utmost importance. This literature review thus highlights the need for sharing best practices, especially in the policy arena, to benefit decentralized settings in LMICs. According to the World Bank, 45 countries in Africa are classified as either low income or lower middle income [51]. The review identified cervical cancer screening implementation reports in decentralized settings in only eight African countries. Thus, at the time of the search, 37 low- or lower middle-income African countries had no reports that fit our inclusion criteria. Most of the included papers (89%) were published more recently (2010 to 2015), showing growing attention to this topic. However, the overall lack of published reports in decentralized low-resource settings within low-income African countries highlights a need to evaluate the implementation of visual inspection cervical cancer screening implementation in all settings to facilitate the sharing of best practices.

Despite no papers from the review having an explicit policy focus, the included articles did describe findings with notable policy implications, in particular cervical cancer screening implementation barriers. We identified an equal number of articles (n = 17, 89%) describing supply-side barriers to those describing demand-side barriers. However, when evaluating the number of barrier mentions, we identified nearly twice as many demand-side barrier mentions (82) as compared to supply-side mentions (45) across all papers. This finding reinforces the perspective that the successful implementation of a visual inspection cervical cancer screening program requires careful consideration of the local context into which the health service is being implemented as well as the need for robust engagement of individuals and the community as a whole. There is a current lack of implementation science projects reporting best practices and the barriers to effective cervical cancer prevention and control programs in these community health systems. These are some of the most marginalized communities in the world. Research capacity may be lacking even where the development of new programs is ongoing. Given this disparity, the development and research communities should align more robustly to build services and evidence where they are most needed.

Using the Levesque framework, we identified an even spread of reported demand-side barriers across the access categories. Supply-side barrier reports, however, were concentrated within three specific areas: the most reports were from Availability and Accommodation, followed by Approachability, and, finally, Affordability. Community health systems in rural Africa may be able to strengthen their underperforming services or apply new principles to expand current services to other locations with a focus on the policy-relevant recommendations of this review. These salient points address the most common problems regarding the implementation of cervical cancer visual inspection screening service and sustainability in similar contexts.

### Policy relevance of findings for the Senegal partnership

The literature lacks policy-specific papers focused on the decentralized low-resource settings in Africa. Reflecting the findings of this literature, since 2014, the Senegal partnership has encountered many barriers. We have used the analysis of this literature review to develop and contextualize further steps in our implementation plan. As a result, we have developed two key initiatives to address many of these contextual challenges. The intent, in upholding the principles of the Dynamic Sustainability Framework [52], is to strengthen the local health system through establishing inherent, iterative processes of responsiveness to changing contexts. In so doing, we hope to facilitate appropriate and timely adaptation of the local health service policy and, ultimately, ensure the sustainability of the screening program. For this reason, we are reporting the policy relevance of the findings of this literature review specifically on the two key strategic implementation initiatives to illustrate how the findings of this review have been applied within the specific context of our described partnership.

### Initiative 1: addressing determinants of uptake and behavioral intervention

One of the partnership’s most significant challenges has been increasing screening uptake among the highest risk women [28]. The estimated participation rate for cervical cancer screening in Senegal (where incidence peaks between the ages of 45 and 54) is very low, nationwide – 1.9% for women ages 40 to 49 and 0% for women over the age of 50 – because of the lack of accessible screening services [53]. Given that in Senegal, HPV positivity is increased in older-age women (a 2002 study of 1639 women showed that 8.6% of women ages 55–59 are high-risk HPV positive) [54], screening in this age range is critical.

Our first principal initiative is, therefore, centered around a robust investigation of the determinants of cervical cancer screening uptake and sustained utilization in this region. The information from this review guided the development and refinement of a context-specific peer education behavioral intervention to improve screening uptake. To inform the participatory development of this program, we have assessed barriers and facilitators of screening at multiple levels: individuals (women aged 30 to 59), households (family or principal social unit of at-risk women), and the community (immediate village or neighborhood with common amenities of at-risk women). We hypothesize that a peer education program (implemented through Care Groups [55,56]) that adapts to changing contexts over time and is targeted at a multi-level audience will result in early, widespread uptake and sustained use of the VIA cervical cancer screening program. This hypothesis has yet to be fully tested. We are also evaluating the intervention’s impact on reducing stigma surrounding cervical cancer. Study findings continue to inform programmatic planning in the Kedougou Region, and the peer education curriculum we have developed may serve as a template for maximizing early impact of new cervical cancer screening services implemented in other areas of rural Senegal. The long-term goal with these data is to inform national-level policy to guide the implementation of cervical cancer screening programs in other rural Senegal regions (total rural population of 8.8 million [57]) with no current access to cervical cancer screening services.

### Initiative 2: addressing workforce attrition and sustained midwifery training

Another major barrier that has shaped the strategy for ensuring the long-term sustainability is the considerable attrition rate among midwives in the region. Because Kedougou is very underdeveloped, with limited infrastructure, health care personnel routinely leave after two to five years to work in areas closer to the capital city of Senegal. Of the original 63 health care workers trained by the end of 2013, only 19 remained in the region at the end of 2015, an attrition rate of 70%. By the end of 2017, an additional 24 midwives were transferred out of the region. Because midwives do not currently learn VIA screening skills through their formal training, all new midwives posted into the region must receive this in-service training. There are often delays, leaving accessibility of the screening service often unreliable.

To respond to this considerable challenge, the partnership’s second principal initiative is to collaborate with the midwifery training center supported by the Senegal Ministry of Health and Social Action in the neighboring region of Tambacounda. With multiple components of the intervention being informed by this literature review, we will establish VIA screening and cryotherapy procedural skills as part of the standard pre-service training curriculum. This will also ensure reliable access to high-quality training for midwives in Kedougou and other surrounding regions. All midwives will also be taught a cervical cancer screening health services quality improvement process based on EngenderHealth’s COPE® approach [25–27] and to develop basic skills in implementation research. Through an ongoing quality improvement process at the primary health care facility level and developing research capacity in the region, we hope that health service policy will be informed by the local context and will be adaptable as future challenges arise.

### Limitations

This literature review is focused on a specific topic (visual inspection cervical cancer screening) within a specific context (policy relevance in rural decentralized regions of Africa). Furthermore, given that partner types, contexts, and objectives differ considerably across global health partnerships, we have attempted to aggregate the reported barriers across all papers into a policy narrative that is most relevant to the Senegal–Peace Corps–UIC partnership’s specific purposes. Therefore, the generalizability of these findings is limited. In addition, the knowledge gained from this literature review was applied to the partnership strategic planning at the time. We are publishing this information some time after the occurrence of this exercise to illustrate how this knowledge helped inform the direction of our own partnership. As such, more recently published literature is not contained within this review. However, the implementation science implications within our partnership and context hold significant value and, therefore, this shortcoming does not lessen the value of the primary lessons gained through this review and application of key findings. This report may provide value, lessons learned, and best practices for others confronting questions of ongoing barriers and ultimate programmatic sustainability for implementation of cervical cancer screening health services.

## Conclusions

Cervical cancer prevention requires a multi-pronged approach including both primary and secondary prevention through accessible vaccination and screening programs [3]. Given that access to vaccination in many LMICs remains limited by considerable systems-level constraints, comprehensive cervical cancer prevention and control in low-resource settings requires a coordinated focus on building accessible and low-cost screening programs. To achieve this goal, advancements in implementation research in this area and a keen understanding of how to adapt evidence-based solutions to local contexts are critical. The affordable screening technique using visual inspection has demonstrated sound evidence of effectiveness for decades. Nonetheless, adoption of this cervical cancer screening technique into local contexts has been limited, resulting in continued absence or limited access in many low-resource settings globally. Through this review, the local context-informed reflection and the careful consideration of the reported lessons learned in similar external contexts have been critical in shaping our partnership’s interventions aimed at addressing challenges to sustainability. This contextual literature review has, therefore, proven informative in shaping cervical cancer screening policy and strategic planning in Senegal. As additional cervical cancer screening technologies are developed in the coming years, these new approaches may contribute to the refinement of clinical algorithms guiding cervical cancer prevention and control programs. The assurance of quality and the long-term sustainment of newly implemented cervical cancer screening programs necessitate considerable attention to systems-level factors beyond the development of technologies and building health workforce capacity. The establishment of high-quality, culturally appropriate, effective cervical cancer screening programs requires adequate supply chains, reliable referral systems, registries, and tracking systems that ensure timely follow-up, quality assurance provision, robust person- and family-centered outreach programs, and governance systems that facilitate responsiveness to capacity challenges such as workforce attrition, among many other functional requirements.

We are hopeful that others attempting to implement or strengthen cervical cancer visual screening services in decentralized areas of Africa will also recognize the findings of this review to be helpful as a construct for identifying evidence for potential barriers, recommendations for overcoming them, and informing policy for tailoring their approaches for optimal cervical cancer screening and control program development and sustainability. This is imperative for addressing a preventable cancer which has deadly consequences for women, families, and societies when left unscreened and untreated.

## Paper context

In 2014, a partnership involving a rural region of Senegal was facing cervical cancer screening service sustainability barriers and began adapting regional-level policy to address implementation challenges. This literature review empowered the partnership to develop a strategic plan to address major structural challenges including workforce retention and client uptake, among others. This article reports the literature review as well as a narrative describing the evolution of the service implementation and policy adaptation since 2014.

## Appendix Appendix: PubMed Search Conducted 11/30/2014

Three duplicates were removed from 2698 citations after moving them from PubMed to RefWorks - bringing the total citations to 2695.

Note, duplication of this search at a later date is more closely approximated using two date filter to compensate for the ongoing post-search expansion of PubMed**: (“1950/01/01„[Date - Create] : “2014/11/30„[Date - Create]) AND (“1950/01/01„[Date - Publication] : “2014/11/30„[Date - Publication])**

***High yield search***

1/

((“Uterine Cervical Neoplasms„[Mesh] OR “Uterine Cervical Dysplasia„[Mesh] OR “Cervical Intraepithelial Neoplasia„[Mesh] OR ((Cervical OR cervix OR cervixes OR cervices OR exocervix OR exocervical OR exocervices OR endocervix OR endocervices OR endocervical OR ectocervix OR ectocervices OR ectocervical OR “Cervix Uteri„[Mesh] OR cervicovaginal) AND (cancer[tw] OR cancers[tw] OR malignancy[tw] OR malignancies[tw] OR dysplasia OR dysplasias OR neoplasia OR neoplasias OR neoplasm OR neoplasms OR neoplastic OR lesion OR lesions OR abnormal OR abnormalities OR adenocarcinoma OR adenocarcinomas OR precancerous OR precursor OR warts OR tumor[tw] OR tumors[tw] OR tumour[tw] OR tumours[tw]) OR HPV OR human papillomavirus OR “Papillomavirus Infections"[Mesh]))) AND ((((("Acetic Acid"[Mesh] OR “acetic acid”[tw] OR iodine OR Lugol's OR Lugol) AND ((VIA AND visual) OR (VILI AND visual) OR “visual inspection” OR “visual screening” OR “visual observation” OR “visual assessment” OR “visual identification” OR “visual recognition” OR “visual detection” OR “visual evaluation” OR “naked eye” OR “naked-eye”)))))

***Low yield search (multi-faceted)***

2/

“Uterine Cervical Neoplasms"[Mesh] OR “Uterine Cervical Dysplasia"[Mesh] OR “Cervical Intraepithelial Neoplasia"[Mesh] OR ((Cervical OR cervix OR cervixes OR cervices OR exocervix OR exocervical OR exocervices OR endocervix OR endocervices OR endocervical OR ectocervix OR ectocervices OR ectocervical OR “Cervix Uteri"[Mesh] OR cervicovaginal) AND (cancer[tw] OR cancers[tw] OR malignancy[tw] OR malignancies[tw] OR dysplasia OR dysplasias OR neoplasia OR neoplasias OR neoplasm OR neoplasms OR neoplastic OR lesion OR lesions OR abnormal OR abnormalities OR adenocarcinoma OR adenocarcinomas OR precancerous OR precursor OR warts OR tumor[tw] OR tumors[tw] OR tumour[tw] OR tumours[tw]) OR HPV OR human papillomavirus OR “Papillomavirus Infections"[Mesh])

3/

test[tw] OR testing[tw] OR tests[tw] OR tested[tw] OR screen[tw] OR screening[tw] OR screenings[tw] OR screened[tw] OR prevention[tw] OR preventions[tw] OR preventive[tw] OR preventing[tw] OR prevent[tw] OR prevents[tw] OR campaign[tw] OR campaigns[tw] OR campaigning[tw] OR “health promotion"[tw] OR “health promotions"[tw] OR “early detection"[tw] OR “early diagnosis"[tw] OR “early diagnoses"[tw] OR “early medical intervention"[tw] OR “early medical interventions"[tw] OR “early intervention"[tw] OR “early interventions"[tw] OR vaginal smear*[tw] OR cervical smear*[tw] OR “Mass Screening"[Mesh] OR “Preventive Health Services"[Mesh] OR “Preventive Medicine"[Mesh] OR “Primary Prevention"[Mesh] OR “Early Detection of Cancer"[Mesh] OR “Early Diagnosis"[Mesh] OR “Early Medical Intervention"[Mesh] OR “prevention and control"[Subheading] OR “Health Promotion"[Mesh] OR “Gynecological Examination"[Mesh] OR “Diagnostic Techniques, Obstetrical and Gynecological"[Mesh]

4/

("Africa"[Mesh] OR Africa OR African OR Sahara OR Sub-Saharan OR subSaharan OR “Africa South of the Sahara"[Mesh] OR “Southern Africa” OR “Africa, Central"[Mesh] OR “Africa, Eastern"[Mesh] OR “Eastern Africa” OR “Africa, Southern"[Mesh] OR “Africa, Western"[Mesh] OR “Western Africa” OR “Western Sahara” OR “Africa, Northern"[Mesh] OR “North Africa” OR “Northern Africa” OR “Sahrawi Arab Democratic Republic” OR Sahrawi OR Algeria OR Algerian OR Egypt OR Egyptian OR Libya OR Libyan OR Morocco OR Moroccan OR Tunisia OR Tunisian OR Cameroon OR Cameroonian OR “Central African Republic” OR Chad OR Chadian OR Congo OR “Congo Brazzaville” OR “Democratic Republic of the Congo” OR Congolese OR “Equatorial Guinea” OR “Equatorial Guinean” OR Gabon OR Gabonese OR Burundi OR Burundian OR Djibouti OR Djiboutian OR Eritrea OR Eritrean OR Ethiopia OR Ethiopian OR Kenya OR Kenyan OR Rwanda OR Rwandian OR Rwandan OR Somalia OR Somalian OR Somali OR Somaliland OR Sudan OR Sudanese OR “South Sudan” OR Tanzania OR Tanzanian OR Uganda OR Ugandan OR Angola OR Angolan OR Botswana OR Botswanan OR Lesotho OR Basotho OR Malawi OR Malawian OR Mozambique OR mozambican OR Namibia OR Namibian OR “South Africa” OR “South Africa"[Mesh] OR “South African” OR Swaziland OR Swazi OR Zambia OR Zambian OR Zimbabwe OR Zimbabwean OR Benin OR Beninian OR ”Burkina Faso” OR “Cape Verde” OR “Cape Verdian” OR “Cote d'Ivoire” OR Gambia OR Gambian OR Ghana OR Ghanaian OR Guinea OR Guinean OR “Guinea-Bissau” OR “Guinea-Conakry” OR Liberia OR Liberian OR Mali OR Malian OR Mauritius OR Mauritania OR Mauritanian OR Niger OR Nigerien OR Nigeria OR Nigerian OR Senegal OR Senegalese OR “Sierra Leone” OR “Sierra Leonean” OR Togo OR Togolese OR Afr OR “Developing Countries"[Mesh] OR “medical missions"[tw] OR “official medical missions"[tw] OR “Medical Missions, Official"[Mesh] OR ngo[tw] OR ngos[tw] OR “non-governmental organizations”[tw] OR “emerging countries”[tw] OR “emerging nations”[tw] OR “emerging economies”[tw] OR “emerging economy”[tw] OR “low resource countries”[tw] OR “low resource nations”[tw] OR “low resource regions”[tw] OR “low resource region”[tw] OR “low resource setting”[tw] OR “low resource settings”[tw] OR “underdeveloped countries"[tw] OR “underdeveloped country”[tw] OR “poorest countries”[tw] OR “poorest nations”[tw] OR “impoverished nations”[tw] OR “impoverished nation”[tw] OR “developing nation”[tw] OR “developing nations”[tw] OR “developing countries”[tw] OR “developing nation”[tw] OR “less developed nation"[tw] OR “less developed nation”[tw] OR “less developed country”[tw] OR “less developed countries”[tw] OR “least developed nations"[tw] OR “least developed country”[tw] OR “least developed countries”[tw] OR “third-world”[tw] OR multi-country[tw] OR multi-national[tw] OR “emerging economy countries"[tw])

5/

("Neck"[Mesh] OR neck OR “head and neck” OR “Cervical Vertebrae"[Mesh] OR “cervical vertebrae” OR “cervical plexus” OR “cervical atlas” OR “neck pain” OR “Esophageal Diseases"[Mesh] OR “Spinal Diseases"[Mesh] OR “Spinal Cord Diseases"[Mesh] OR transcervical OR lumbosacral OR lumbar OR thorax OR thoracic OR larynx OR “disc”[tw] OR discs OR arthroplasty OR osteoporotic OR discectomy OR myelopathy OR “Mycobacterium Infections"[Mesh] OR tuberculin OR tuberculosis OR tuberculomas OR tuberculoma OR “root caries” OR “dental caries” OR dentin OR “tooth cervix” OR dentition OR “thyroid diseases” OR “Thyroid Diseases"[Mesh] OR thyroid[ti] OR parathyroid OR “thyroid cancers” OR “thyroid cancer” OR “thyroid neoplasms” OR “thyroid malignancies” OR “thyroid nodules” OR “Thyroid Neoplasms"[Mesh] OR thyroidectomy OR “ectopic pregnancies”[ti] OR “ectopic pregnancy”[ti] OR preterm birth*[ti] OR preterm deliver*[ti] OR premature rupture*[ti] OR fetal[ti] OR caesarean[ti] OR “Abortion, Induced"[Majr] OR abortion*[ti] OR “cervical cerclage” OR “cervical ripening” OR “cervical incompetence” OR fistula OR “Obstetric Labor Complications"[Majr] OR “obstetric complications”[ti] OR “labor complications”[ti] OR “African-American”[tw] OR lions OR cows OR bovine OR bovines OR cattle OR heifers OR chimpanzees OR pigs OR “guinea-pigs” OR mice OR rats OR cats OR dogs OR rabbits OR zebrafish OR hamsters)

6/

#2 AND #3 AND #4

7/

#6 NOT #5 (Completes the Low Yield retrieval set)

***Combine the Low Yield and High Yield searches with English filter and date limits***

8/

#7 OR #1 (Order is important)

9/

#8 AND ("1950/01/01"[Date - Create] : “2014/11/30"[Date - Create]) AND ("1950/01/01"[Date - Publication] : “2014/11/30"[Date - Publication]) AND English[lang]
